# Innovations in Advanced Endoscopic Resection of Early Upper Gastrointestinal Cancer

**DOI:** 10.3390/jcm15124530

**Published:** 2026-06-11

**Authors:** Andrea Sorge, Pieter Jan Poortmans, Michele Montori, Maria Eva Argenziano, Edoardo Vincenzo Savarino, David J. Tate

**Affiliations:** 1 Department of Surgery, Oncology and Gastroenterology, University of Padua, 35128 Padua, Italy; andrea.sorge@unipd.it (A.S.); edoardo.savarino@unipd.it (E.V.S.); 2 Gastroenterology Unit, Azienda Ospedale-Università Padova, 35128 Padua, Italy; 3 Department of Gastroenterology and Hepatology, University Hospital Ghent, 9000 Ghent, Belgium; pieterjan.poortmans@uzgent.be (P.J.P.); mariaeva.argenziano@gmail.com (M.E.A.); 4 Faculty of Medicine and Health Sciences, Ghent University, 9000 Ghent, Belgium; 5 Department of Gastroenterology & Hepatology, University Hospital Brussels (UZ Brussels), 1090 Brussels, Belgium; 6 Clinic of Gastroenterology, Hepatology and Emergency Digestive Endoscopy, Università Politecnica delle Marche, 60126 Ancona, Italy; michele.montori@ospedaliriuniti.marche.it

**Keywords:** endoscopic submucosal dissection, endoscopic resection of gastric cancer, oesophageal cancer, traction, endoscopic full-thickness resection, therapeutic endoscope, REACT, intermuscular dissection, STER, non-curative resection, artificial intelligence

## Abstract

Endoscopic resection (ER) has become the preferred curative-intent treatment for early upper gastrointestinal cancer, given its superior safety profile compared to surgery. Over the past decade, technological and procedural innovation has substantially expanded the scope, safety, and precision of endoscopic submucosal dissection (ESD) and related techniques. This review synthesises current evidence on key advances relevant to upper gastrointestinal ESD practice. Enhanced imaging modalities have improved lesion detection and characterisation, as well as recognition of intraoperative anatomical structures during third-space endoscopy. A new generation of therapeutic endoscopes combines high-definition optics with substantially improved tip-down angulation and channel size, addressing a longstanding gap between diagnostic-class image quality and procedural capability. Resection strategies—including mechanical traction systems, saline immersion therapeutic endoscopy (SITE), and luminal drainage techniques—have reduced procedural complexity and improved dissection conditions. Dedicated closure technologies have improved management of large resection defects, potentially reducing resection-related morbidity. Deep resection techniques, including submucosal tunnelling endoscopic resection (STER), device-assisted endoscopic full-thickness resection (FTRD), knife-assisted full-thickness resection (kFTR), and endoscopic intermuscular dissection (EID), are extending organ-preserving resection to deeply invasive cancers and subepithelial lesions. Management of non-curative ESD resections is being refined through multicentre risk stratification studies. Advances in simulation, competency-based training, and artificial intelligence hold promise for standardising technique acquisition and real-time procedural support. Together, these innovations are reshaping upper gastrointestinal oncology by positioning minimally invasive, organ-preserving digestive endoscopy as a central therapeutic strategy.

## 1. Introduction

Early detection of upper gastrointestinal (UGI, oesophageal and gastric) cancer has increased significantly in recent years, driven by advances in high-definition endoscopy, endoscopy training programs, structured endoscopic surveillance programmes, and population-based screening in high-incidence regions [1,2]. As detection shifts toward mucosal and superficial submucosal lesions, endoscopic resection (ER), and endoscopic submucosal dissection (ESD) in particular, has become the standard of care for curative-intent treatment of appropriately staged early (T1) UGI cancers. ESD enables en bloc resection with accurate histopathological staging, preserving gastric and oesophageal function while avoiding the morbidity of surgical resection in case of curative excision [1,2].

Despite its oncological efficacy, ESD remains technically demanding. Procedure time is substantial and often requires general anaesthesia. Complication rates, particularly delayed bleeding and perforation, require vigilant patient management, and the learning curve is prolonged in Western practice compared to Eastern countries [3]. Over the past decade, a wave of technological and procedural innovation has addressed many of these challenges. Image enhanced endoscopy (IEE) has improved pre-procedural lesion detection and characterisation. Purpose-designed therapeutic endoscopes, mechanical traction devices, and novel immersion techniques have improved visualisation and recognition of anatomical structures, reducing operative complexity. Dedicated closure systems can help prevent post-procedural adverse events. Advanced resection strategies, including submucosal tunnelling endoscopic resection (STER), endoscopic intermuscular dissection (EID), and knife-assisted full-thickness resection (kFTR), have expanded the boundaries of endoscopic organ preservation. Simultaneously, advances in simulation-based training and artificial intelligence (AI)-guided procedural support are beginning to lower the barrier to widespread adoption.

This narrative review summarises the current evidence on key innovations in upper gastrointestinal endoscopic resection, emphasising advances that are already being incorporated into clinical practice or show strong near-term potential for adoption.

This review is intended for endoscopists, gastroenterologists, and surgeons involved in the curative-intent management of early upper gastrointestinal cancer, and for trainees developing advanced resection skills. Its scope is deliberately confined to therapeutic innovation in endoscopic resection of early (T1) oesophageal and gastric neoplasia. A focused reappraisal is timely because several of the developments discussed—adaptive multipoint traction, saline-immersion dissection, dedicated closure platforms, deep-resection techniques such as EID and kFTR, and competency-based training and artificial intelligence support—have emerged or matured only in the last few years and are not yet consolidated in current society guidelines. By mapping these advances onto everyday upper gastrointestinal practice, this review clarifies the current level of evidence supporting each technique and highlights where prospective data are still required.

## 2. Methods

A structured literature search was performed in MEDLINE (PubMed), EMBASE, and the Cochrane Library, using combinations of MeSH terms and free-text keywords covering endoscopic submucosal dissection, endoscopic full-thickness resection, submucosal tunnelling endoscopic resection, image-enhanced endoscopy, traction and immersion techniques, endoscopic closure, ESD training, and artificial intelligence in endoscopy. Searches were updated up to February 2026 and limited to publications in English. International society guidelines and references cited in the retrieved articles were also screened. The authors selected the studies discussed in this narrative review.

Generative AI tools were used solely for language editing during the preparation of this manuscript. The authors carefully reviewed and edited the text and remain fully responsible for all content, interpretations, and conclusions presented in the paper.

## 3. Image-Enhanced Endoscopy for Lesion Detection, Characterisation, and Margin Control

**Figure 1:**
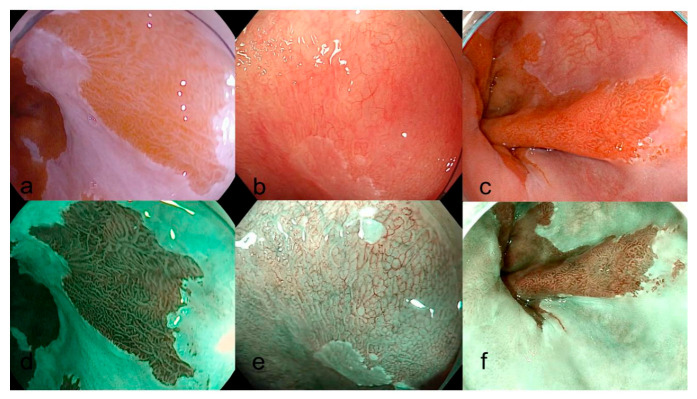
Endoscopic appearance of Barrett’s oesophagus (BE) across different high-definition platforms and image-enhanced endoscopy (IEE) modalities. (**a**) BE under white-light imaging (WLI). (**b**) BE under WLI. (**c**) BE under WLI. (**d**) BE under i-SCAN (same lesion as (**a**)). (**e**) BE under narrow-band imaging (NBI; same lesion as (**b**)). (**f**) BE under blue-light imaging (BLI; same lesion as (**c**)). Abbreviations: BE, Barrett’s oesophagus; BLI, blue-light imaging; NBI, narrow-band imaging; WLI, white-light imaging.

Accurate pre-procedural characterisation of early UGI neoplasia, including determination of histological type, invasion depth, and lateral extent, is fundamental to ESD planning. Image-enhanced endoscopy (IEE) has become integral to this process, with innovations organised here by the clinical problem they address rather than by manufacturer platform (Figure 1).

### 3.1. Lesion Detection

Detection of early UGI neoplasia remains the critical first step. Several platform-specific imaging modalities have been evaluated for lesion detection in UGI endoscopy.

Linked colour imaging (LCI; Fujifilm, Tokyo, Japan) uses post-processing image enhancement to highlight colour differences between normal and abnormal mucosa. The LCI-FIND multicentre RCT (N = 1502) demonstrated that LCI significantly improved detection of gastric neoplasms compared with white-light imaging (WLI), with a relative risk of 1.67 (95% CI 1.12–2.49) [4]. A subsequent meta-analysis encompassing 11 studies and 7836 patients confirmed that LCI consistently improved gastric neoplasm detection compared with WLI [5]. Blue-light imaging in its bright mode (BLI-bright; Fujifilm), which uses short-wavelength illumination to enhance mucosal surface and vascular contrast, improved the real-time detection rate of early gastric cancers in a dedicated RCT (N = 629), with sensitivity reaching 93.1% compared with 50.0% for WLI [6].

Texture and colour enhancement imaging (TXI; Olympus, Tokyo, Japan) applies digital image processing to enhance mucosal microstructure and colour gradation. Despite its theoretical advantages, a multicentre RCT (Kadota et al., 2024) found that TXI did not significantly outperform WLI for the detection of early gastric neoplasms [7]. This is an important finding that tempers enthusiasm for TXI as a detection tool.

Narrow-band imaging (NBI; Olympus), the long-established optical enhancement modality on this platform, shows a similar site-specific pattern. A multicentre RCT in high-risk patients found no significant improvement in early gastric cancer detection with second-generation NBI over WLI [8], whereas a separate multicentre RCT demonstrated that NBI significantly improved detection of superficial squamous cell carcinoma (SCC) in the oesophagus and head and neck region compared with WLI [9]. For oesophageal SCC screening, NBI achieves equivalent sensitivity and superior specificity compared with Lugol chromoendoscopy, without the discomfort and bronchospasm risk of iodine application [10]. This detection benefit in the oesophagus for SCC but not for gastric adenocarcinoma likely reflects the sharper vascular contrast signatures of early oesophageal SCC against normal squamous mucosa. NBI’s primary role in the UGI tract, particularly its use in lesion characterisation, is discussed in Section 3.2.

i-Scan and i-Scan optical enhancement (i-Scan OE; Pentax Medical, Tokyo, Japan) provide the third major commercial approach. Conventional i-Scan applies real-time digital post-processing in three modes, surface enhancement (SE), contrast enhancement (CE), and tone enhancement (TE), whereas i-Scan OE adds optical bandpass pre-filters that narrow the illumination spectrum to highlight superficial vasculature, making it conceptually analogous to NBI and BLI [11]. A recent systematic review and meta-analysis demonstrated improved diagnostic performance of i-Scan over WLI for early gastric cancer detection [12].

### 3.2. Lesion Characterisation

Characterisation of mucosal and vascular microarchitecture is critical for determining invasion depth and guiding the decision between endoscopic and surgical management.

NBI enhances surface and vascular patterns using filtered wavelengths. NBI-based magnifying endoscopy for characterisation of early oesophageal SCC has demonstrated high diagnostic accuracy in meta-analysis [13], and remains the reference standard for ESCC characterisation using the magnifying endoscopic classification of the Japan Esophageal Society evaluating the intrapapillary capillary loop (IPCL) [14]. In Barrett’s oesophagus, NBI has demonstrated high accuracy for identification of high-grade dysplasia and specialised intestinal metaplasia, with a meta-analysis reporting sensitivity of 96% and specificity of 94% for high-grade intraepithelial neoplasia [15]. For gastric neoplasia, NBI magnification enables detailed mucosal and vascular assessment using the vessel-plus-surface (VS) classification system, achieving very high diagnostic accuracy, sensitivity, and specificity for differentiation of neoplastic from non-neoplastic mucosa in prospective multicentre evaluation, with the caveat that performance is attenuated for pale mucosal lesions such as signet-ring-cell carcinoma [16]. The MESDA-G algorithm subsequently provided a simplified, stepwise diagnostic framework derived from the VS classification to facilitate its adoption in routine practice [17].

BLI provides analogous vascular and surface pattern enhancement for gastric and oesophageal lesion characterisation. Amber-Red Color Imaging (ACI; Fujifilm), a more recently introduced IEE modality, enhances tissue contrast, allowing easier identification of key anatomical structures (i.e., vessels and muscularis propria) during third-space endoscopy. A prospective cohort study including ESD and peroral endoscopic myotomy (POEM) procedures demonstrated that ACI provided improved visualisation of the submucosal dissection plane, vascular structures, and fascial layers compared with WLI [18].

i-Scan OE (Pentax), is a virtual chromoendoscopy technology that uses selected light wavelengths to enhance the contrast of superficial mucosal and vascular patterns; when combined with magnification, this improves lesion characterisation.

### 3.3. Improved Magnification and Depth of Focus

Maintaining sharp optical focus across varying working distances is a practical challenge during ESD, where the endoscope-to-tissue distance changes continuously during flap elevation and dissection.

Each major manufacturer offers a different solution to this problem. On the Olympus platform, Dual Focus endoscopy enables switching between standard and near-focus magnification without additional optical components. In a randomised trial, Dual Focus combined with NBI improved detection and characterisation of superficial squamous neoplasia in the pharynx and oesophagus [19]. Extended depth-of-field (EDOF) optics, incorporated in the EVIS X1 EZ-1500 series, broaden the range of sharp focus without requiring the operator to actively switch between focal planes, maintaining tissue clarity across varied working distances simultaneously, which is of potential value in therapeutic endoscopy for maintaining optical clarity during dissection.

Fujifilm has historically addressed this requirement with optical zoom magnifying gastroscopes, including the long-standing 760EZ-series zoom platform, which provides true mechanical optical zoom for high-resolution mucosal and vascular assessment at close range. Pentax similarly offers true optical zoom through dedicated magnifying gastroscopes (e.g., the EG29-i10c), and its OptiVista platform combines this magnification capability with i-Scan OE pre-filters for integrated enhanced visualisation [20]. Each of these approaches—Dual Focus, EDOF, or true optical zoom—represents a different engineering route to the same clinical goal of high-fidelity close-range visualisation during ESD planning and intraoperative margin assessment.

In the context of ESD itself, all IEE and magnification modes serve to confirm the lateral extent of lesions, guide marking, and identify residual neoplastic tissue at the margins intraoperatively. This is particularly relevant for oesophageal SCC, Barrett’s-associated adenocarcinoma and flat gastric lesions, where macroscopic extent may be difficult to delineate under WLI. Furthermore, AI-based computer-aided detection (CADe) and characterisation (CADx) systems are being developed and studied for both oesophageal and gastric neoplasia, and a dedicated treatment of this literature is beyond the scope of the present review. The image-enhanced and magnification modalities relevant to the ESD workflow, with the strength of evidence supporting each, are summarised in Table 1.

## 4. A New Generation of Therapeutic Endoscopes

Until recently, instrument choice in therapeutic upper GI endoscopy has involved an awkward trade-off between three imperfect options. Standard diagnostic gastroscopes such as the EZ-1500 series or the 760EZ zoom platform deliver the highest-quality optics in UGI endoscopy but handicap therapeutic work through limited tip-down angulation (which restricts access to lesions behind the pylorus, in the gastric fundus, or at the cardia) and small working channels (typically 2.0–2.8 mm) that constrain suction of blood or fluid (especially when an accessory, like an ESD knife, is inserted down the working channel) and limit accessory choice. Standard large-diameter therapeutic gastroscopes—with 11.5–12.6 mm insertion tubes and 3.7–3.8 mm channels—offer the suction and accessory capacity needed for complex dissection but at a notable cost: decreased retroflexion ability, reduced manoeuvrability in tight or angulated anatomy, and substantially poorer optical performance than their slimmer diagnostic counterparts. Narrow-calibre scopes, conversely, offer a distinct procedural advantage in tight working spaces such as submucosal tunnelling endoscopic resection (STER), where a slim insertion tube manoeuvres comfortably inside a confined tunnel without compressing the mucosal flap.

The newest generation of therapeutic instruments addresses these constraints directly. The ELUXEO^®^ EG-840T (Fujifilm) is a normal-diameter therapeutic gastroscope with enhanced downward angulation to 160° (from 90° on previous models) while maintaining an upwards angulation of 210°, enabling access to difficult lesions. The EG-840TP is a super-slim therapeutic endoscope combining a 7.9 mm insertion tube with a 3.2 mm working channel [21]. The optics on both scopes are high-definition rather than zoom-quality, marking a clear step forward in image quality for therapeutic instruments and narrowing the gap between diagnostic and therapeutic platforms.

These scopes have been evaluated in POEM (with equivalent outcomes to conventional therapeutic scopes in a propensity-matched study [22]), Barrett’s oesophagus ESD under saline immersion [23], and oesophageal ESD with concurrent nasogastric drainage [24]—the latter relying on the 840TP slim insertion tube to allow co-passage with an naso-gastric tube (NGT).

## 5. Resection Strategies: Traction, Immersion, and Luminal Drainage

Adequate tissue exposure is one of the most determinative factors for ESD procedural efficiency and safety. Several complementary strategies that have been recently described and evaluated in clinical practice address this challenge (Table 2).

### 5.1. Saline Immersion Therapeutic Endoscopy (SITE)

The acronym SITE (saline immersion therapeutic endoscopy) describes the technique of filling the lumen with isotonic saline to create an immersion environment during ESD [25,26]. SITE is specifically distinguished from earlier “water-pocket” or “underwater” techniques by the mandatory use of isotonic saline rather than water. This distinction is clinically significant: saline’s electrolyte content enhances current conduction, enabling effective coagulation at lower diathermy settings; isotonic fluid preserves tissue integrity (avoiding the friability caused by hypotonic water); and ESD knives dissect more efficiently in saline than in water [26].

The saline column provides several physiological advantages: buoyancy of the elevated mucosal flap (often reducing or eliminating the need for traction), improved optical clarity through a magnification effect, haemostatic pressure, and elimination of electrosurgical smoke. These properties improve visualisation of the dissection plane, particularly in anatomically challenging segments [25,27]. Saline immersion also reduces gas-related complications by replacing CO_2_ insufflation, decreasing the risk of subcutaneous emphysema and tension capnoperitoneum.

The technique was initially described for gastric ESD, where water-pocket ESD demonstrated improved submucosal visualisation and reduced procedure time [25]. Large Western cohort data have confirmed favourable outcomes for SITE-based colorectal ESD, including low general anaesthesia and hospital admission rates [28].

#### Luminal Drainage During SITE

A barrier to SITE is the accumulation of large volumes of instilled saline, which can lead to fluid overload, lumen distension with reduced endoscope manoeuvrability and patient hypothermia. Furthermore, the gas generated by high-frequency devices creates a gas–fluid interface that requires frequent suction during the procedure. Wrapping a nasogastric tube (NGT) in a spiral fashion around the endoscope provides concurrent drainage of luminal gas and fluid, minimising the need for frequent aspiration, maintaining the optical benefits of SITE [24]. For oesophageal resections, using a slim gastroscope (7.9 mm) can be useful to enable co-passage with the NGT without reducing manoeuvrability in the narrow oesophageal lumen. Drainage with a 14F, 125-cm nasogastric tube has been described in colorectal case reports as “Asclepius tube” [29,30].

### 5.2. Water Pressure Method for Fibrotic Lesions

The water pressure method (WPM), first described by Yahagi and colleagues, uses directed saline injection to expand the dissection plane and facilitate access to the submucosal space [31]. The WPM provides good countertraction to the submucosal layer and good visualisation of the operative field. Furthermore, it overcomes the effects of gravity during ESD [32]. WPM can be useful in difficult situations such as duodenal ESD and UGI lesions with severe fibrosis from prior treatment attempts, where the standard submucosal cushion cannot be raised. This technique may enable endoscopic retreatment of lesions previously considered beyond endoscopic salvage.

### 5.3. Traction-Assisted ESD

While fluid-based strategies optimise the dissection environment, traction-based approaches address tissue exposure through mechanical counterforce. As noted, the two are not mutually exclusive.

**Evidence base.** Two landmark RCTs established the clinical benefit of traction in UGI ESD. The CONNECT-E trial demonstrated that clip-with-line traction significantly reduced procedure time (44.5 min vs. 60.5 min, respectively; *p* < 0.001) in oesophageal ESD [33]. The CONNECT-G trial showed equivalent benefits in gastric compared to conventional ESD (60.7 vs. 58.1 min, respectively; *p* = 0.45) [34]. Data from meta-analyses consistently confirmed that traction-assisted ESD reduces procedure time (mean difference approximately 15–25 min) and reduces perforation rate compared with conventional ESD, while en bloc and complete resection rates are comparable between the two techniques; the largest effect sizes are observed in oesophageal procedures [35,36,37].

**Single-point traction.** Clip-with-line traction remains widely used given its simplicity and low cost. Spring-loop (SLC) and S-O clip devices provide maintained tension without repeated repositioning. A clip is attached to the resected edge of the specimen and connected to a line that exits through the patient’s mouth, where the operator or assistant can manually adjust the tension during dissection. This external line arrangement allows the magnitude of traction to be modulated throughout the procedure, though the direction of pull is fixed by the clip attachment point and cannot be redirected as the dissection front advances. The principal limitation of all single-point systems remains the inability to fully redirect traction as the dissection front changes orientation, motivating the development of multipoint and adaptive approaches. Furthermore, this strategy is of limited efficacy in large lesions where traction at multiple lesion points is required.

**Multipoint and adaptive traction.** Beyond single-point traction, a family of multipoint and adaptive traction systems has been developed to maintain dynamic counterforce throughout the dissection rather than at a single fixed attachment. These systems share the principle of distributing traction across multiple points on the resection specimen and allowing the operator to redirect or augment force as the dissection front advances.

**Figure 2:**
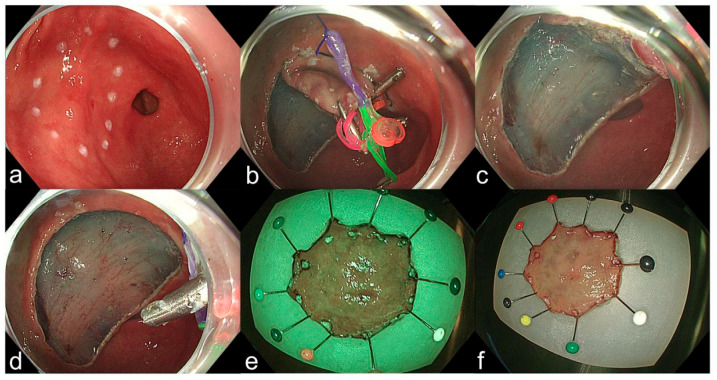
Example of traction-assisted endoscopic submucosal dissection (ESD) of a lesion at the gastric incisura. (**a**) Lesion after circumferential marking. (**b**) Submucosal dissection with improved access provided by the REACT (Repositionable Elastic Adaptive Customizable Traction) system combined with a pulley. (**c**) Progression of the dissection; submucosal exposure remains optimal owing to the adaptive nature of the traction. (**d**) Post-ESD mucosal defect after en bloc resection. (**e**) Resected specimen under narrow-band imaging (NBI). (**f**) Resected specimen under white-light imaging (WLI). Abbreviations: ESD, endoscopic submucosal dissection; NBI, narrow-band imaging; REACT, Repositionable Elastic Adaptive Customizable Traction; WLI, white-light imaging.

The M-loop traction device (MLTD) uses a loop construct anchored across the lesion to provide adjustable bidirectional traction, with consistent reduction in dissection time reported in gastric ESD [38,39]. The ATRACT (Adaptive TRACtion) system, developed by Pioche and colleagues, is a device composed of loops with different functions that can be adjusted to provide increasing traction force as ESD progresses, unlike conventional clip-and-rubber-band methods, in which traction diminishes over time [40]. Prospective multicentre data (50 procedures) demonstrated 100% en bloc resection with the highest reported resection speed for colorectal ESD (61.4 mm^2^/min) [41]. The REACT (Repositionable Elastic Adaptive Customizable Traction) multipoint traction method applies a related concept using multiple rubber bands to create adjustable, distributed countertraction across the specimen (Figure 2) [42,43,44]. A prospective cohort study described the initial experience in colorectal ESD, demonstrating feasibility, adjustability, and effective maintenance of countertraction [45]. REACT’s practical advantages include low material cost, simplicity of construction from widely available components, and ease of intraoperative adjustment. A meta-analysis of rubber band-based traction strategies confirmed efficacy comparable to clip-based methods with the additional advantage of adjustability [46].

To date, the published REACT and ATRACT experience is predominantly colorectal. However, a case report of UGI ESD performed using the REACT has been reported [47].

## 6. Closure Devices and Prevention of Post-Procedural Complications

### 6.1. Novel Through-the-Scope Clips

Standard through-the-scope (TTS) clips remain the first-line closure method for small to moderate mucosal defects after ESD. For larger defects exceeding the span of conventional clips (approximately 10–12 mm), the Mantis clip (Boston Scientific, Marlborough, MA, USA) offers a wider opening and stronger tissue approximation. This novel anchor-pronged clip has the advantage of a strong tissue-grasping capability. Two prospective series have described its application in post-ESD defect closure, reporting high technical success rates and reduced requirement for multiple clips [48,49]. A practical caveat is that the Mantis clip can produce only superficial mucosal apposition if the deployment is not carefully planned: failure to grasp the muscular layer on both sides of the defect risks tenting the mucosa over the wound rather than achieving true layer-to-layer closure, leaving a residual sub-mucosal cavity (submucosal dead space) beneath an apparently closed surface. Operators should aim to engage the muscle bilaterally with each Mantis deployment to avoid this pitfall.

Clip-with-line closure techniques, using nylon line to approximate wound edges before securing with clips, have been developed for large defects, with the reopenable clip over-the-line method (ROLM) showing 100% closure success in a prospective gastric ESD series [50].

### 6.2. Over-the-Scope Clips

Over-the-scope clips (OTSC) provide full-thickness tissue compression and are well-established for closure of ESD perforations, fistulae, and tunnel entry sites after POEM and STER [1]. Its deployment provides reliable transmural sealing with low adverse event rates. While technical success rates are high and adverse event rates low in these indications, OTSC is not typically deployed as a routine first-line defect closure strategy after uncomplicated ESD, where through-the-scope clips or suturing devices are generally preferred (Table 2).

An important caveat for OTSC deployment is the oesophageal location, as this carries a significant risk of iatrogenic luminal stenosis. Its use in the oesophagus should therefore be reserved for situations where the risk of uncontrolled perforation outweighs the stenosis risk.

### 6.3. Endoscopic Suturing

The OverStitch™ (Boston Scientific) endoscopic suturing system enables full-thickness suturing of large resection defects and tunnel entry sites. Two meta-analyses have characterised its performance: one demonstrated high technical success with reduced delayed bleeding and perforation compared with clip-based closure in large defects [51]; the second addressed POEM entry site closure, confirming comparable outcomes to OTSC [52]. OverStitch is particularly applicable to defects exceeding the capacity of clip-based systems.

The SutuArt system (Olympus, Tokyo, Japan) offers an alternative approach for endoscopic hand suturing using a through-the-scope needle holder with barbed dissolving suture, mimicking surgical hand suturing endoscopically. A multicentre pilot study demonstrated feasibility and safety, with zero postoperative bleeding in patients on antithrombotic therapy [53]. First Western experience (19 procedures) confirmed improving technical success with operator experience [54].

X-Tack™ (Boston Scientific) is a through-the-scope suturing system that can facilitate closure of large mucosal defects after ESD or upper gastrointestinal resection, particularly when conventional clips alone are insufficient. By enabling tissue approximation across broad or irregular defects, it may help achieve secure closure and potentially reduce the risk of delayed adverse events.

### 6.4. Stricture Prevention After Circumferential Oesophageal ESD

Post-ESD stricture remains the predominant long-term morbidity risk when oesophageal ESD extends beyond 75% of the luminal circumference. A network meta-analysis comparing prevention strategies found that oral prednisolone, intralesional steroid injection, and biodegradable stents all reduced stricture rates, with oral prednisolone showing the most consistent evidence base [55].

Autologous oral mucosal epithelial cell sheet transplantation, in which cultured epithelial cells are transferred to the post-ESD defect via a temperature-responsive scaffold, dramatically reduced stricture formation in a landmark translational study [56]. This approach remains limited to pioneer centres pending manufacturing standardisation.

## 7. Deep Resection Strategies: EID, STER, and Full-Thickness Resection

### 7.1. Endoscopic Intermuscular Dissection (EID)

Endoscopic intermuscular dissection (EID) is an emerging technique for deep-layer gastric resection in which the dissection plane is developed between the inner oblique and circular layers of the muscularis propria (MP), enabling removal of lesions with superficial MP invasion without full-thickness breach. Anatomically, EID occupies an intermediate position between conventional endoscopic submucosal dissection, which remains within the submucosal plane, and endoscopic full-thickness resection, which is transmural. It therefore has the potential to extend organ-preserving resection to selected cases of deep submucosal or superficial MP invasion (Table 2).

The first description of EID with contemporary instruments was published by Despott et al. in 2024, who demonstrated its feasibility in a small gastric subepithelial lesion and established the intermuscular plane as a discrete anatomical working space [57]. Sorge et al. subsequently reported the first identification of the muscle-retracting sign in the stomach, which allowed adaptive EID of a gastric neuroendocrine tumour with appropriate depth control [47]. The muscle-retracting sign had previously been characterised in the colorectal setting [58] and prospectively validated as a marker of deep submucosal invasion in rectal cancer [59].

**Figure 3:**
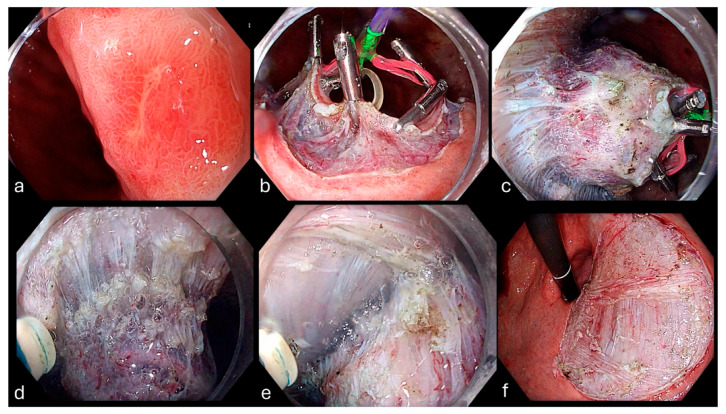
Gastric endoscopic intermuscular dissection of a neuroendocrine tumour. (**a**) Endoscopic appearance of a type 1 neuroendocrine tumour in chronic atrophic gastritis. (**b**) Traction with the REACT system combined with an external surgical wire. (**c**) The oblique muscularis propria layer is exposed. (**d**) Intentional oblique muscularis propria incision to achieve R0 following recognition of a muscle-retracting sign. (**e**) Intermuscular dissection with exposure of the connective tissue in the intermuscular space between the oblique and the circular muscularis propria in the proximal stomach. (**f**) Gastric endoscopic intermuscular dissection defect. Abbreviations: REACT, Repositionable Elastic Adaptive Customizable Traction.

A key anatomical consideration when applying EID to the stomach is that the longitudinal layer of the MP is often too thin and incomplete to reliably prevent full-thickness breach after circular muscularis propria dissection, with an attendant risk of delayed perforation once the defect is exposed to acid within a high-pressure environment. In the proximal stomach, however, the additional oblique layer provides an extra anatomical safeguard: dissection between the oblique and circular layers may leave both the circular and longitudinal layers intact (Figure 3), supporting the feasibility and safety of EID in this region. Prospective series with standardised oncological endpoints are needed to define the clinical role, selection criteria, and long-term outcomes of gastric EID compared to surgical alternatives. Current experience with EID in the upper gastrointestinal tract remains limited to initial case reports from originating groups. Patient selection criteria have not yet been standardised, and long-term oncological outcomes remain undefined. Therefore, EID should currently be considered an experimental technique, with its use restricted to expert centres and preferably within prospective studies or structured registries.

### 7.2. Submucosal Tunnelling Endoscopic Resection (STER)

STER enables en bloc removal of subepithelial lesions (SELs) arising from the muscularis propria (MP) layer of the oesophagus and proximal stomach. By creating a submucosal tunnel 3–5 cm proximal to the tumour and dissecting circumferentially within the tunnel, STER achieves clean MP-layer enucleation while leaving the mucosal entry site intact, preventing mediastinal or peritoneal contamination.

Initial comparative series established STER as superior to video-assisted thoracoscopic surgery (VATS) for oesophageal leiomyomas, with shorter procedure time, lower complication rates, and equivalent en bloc resection rates [60]. Long-term follow-up data confirm durable recurrence-free survival [61]. A recent systematic review and meta-analysis pooling more than 2900 patients reported a pooled R0 resection rate of 92.4%, en bloc resection of 91.5%, a local recurrence rate of 3%, and an acceptable adverse event profile (17.8%) across upper GI applications [62]. Refinement of procedural indication has been guided by the SAFE (Subepithelial-lesion Algorithm for Endoscopic resection) algorithm, which stratifies SEL resectability based on tumour-to-lumen contact angle, infiltration pattern, and depth of MP involvement [58]. STER is best suited to oesophageal, cardia, and fundic SELs with intraluminal or trans-mural growth pattern up to approximately 35 mm; lesions with predominantly extraluminal growth, locations distal to the gastric body where tunnel construction is impractical, or maximum diameter exceeding 35–40 mm are generally outside its scope.

### 7.3. Endoscopic Full-Thickness Resection (EFTR) for Subepithelial Lesions

For SELs that fall outside the indications for STER, particularly antral or distal gastric tumours, those with predominantly extraluminal growth, or small lesions where the cap-and-clip approach is preferable to tunnel construction, endoscopic full-thickness resection (EFTR) provides an alternative. Two main technical strategies exist: device-assisted EFTR using the Full-Thickness Resection Device (FTRD) system, and “classical” or exposed EFTR using freehand ESD-style dissection through the muscularis propria with subsequent defect closure.

**The FTRD system.** The FTRD (Ovesco Endoscopy AG, Tübingen, Germany) consists of a modified over-the-scope clip pre-loaded onto a transparent applicator cap fitted with an integrated 13 mm monofilament snare. Following marking of the lesion, an anchor or grasper is used to retract the lesion fully into the cap; the OTSC is then deployed beneath the lesion to create a serosa-to-serosa seal, and the integrated snare resects the tissue above the clip in a single deployment, achieving full-thickness resection with simultaneous full-thickness closure. The dedicated upper GI variant (“gFTRD”) was introduced from 2017 onwards with a larger-diameter cap and modified delivery system suited to gastric and duodenal anatomy [63]. The lesion size ceiling is approximately 20 mm, dictated by the cap diameter.

The first international multicentre FTRD experience in the upper GI tract reported on a mixed cohort of subepithelial tumours, non-lifting adenomas, and recurrent or scarred lesions across the stomach and duodenum, with technical success of 77% (successful device deployment in 93%), R0 resection of 68% (lower in the duodenum), and adverse events in 21% (predominantly bleeding, with rare delayed perforation; clip-related stenosis where deployment encroached on the pylorus or duodenal lumen); at endoscopic follow-up, no residual or recurrent lesion was found in 30 of 31 patients assessed (97%), although follow-up was short and available for only a subset of the cohort [63]. Dedicated duodenal FTRD series have confirmed feasibility for sub-20 mm duodenal lesions, reporting technical success and R0 resection rates of 80% with no local recurrence at 6-month follow-up, with the duodenum representing the highest-risk site because of wall thinness and proximity to the ampulla [64]. FTRD is not recommended in the oesophagus, where the absence of serosa, tubular geometry, and risk of mediastinitis preclude its safe use. Within the stomach, FTRD is most successful for small antral or distal-body lesions where the cap can engage the wall fully; access to the fundus and high body remains limited by scope angulation with a mounted cap. The principal advantage over surgical wedge resection for small (<20 mm) SELs is the avoidance of laparoscopy and general anaesthetic morbidity; the principal limitation is the size cap, which excludes most lesions otherwise suitable for ESD.

**Classical (exposed) EFTR.** When FTRD is unsuitable, typically for SELs above 20 mm or located outside the antrum/distal body, classical EFTR remains an option in expert centres. The technique combines circumferential ESD-style mucosal incision around the lesion with deliberate full-thickness perforation of the muscularis propria, followed by full-thickness specimen retrieval and defect closure using clips, OTSC, endoloop-clip (“hold and drag”), or endoscopic suturing. Pioneering Chinese series (Zhou and colleagues, Shanghai) demonstrated that classical EFTR for gastric subepithelial tumours arising from the MP achieves a complete resection rate of 100% (26 lesions, predominantly GISTs), with no local recurrence reported, although the mean follow-up was limited to 8 months, and with the intentional full-thickness defects managed entirely endoscopically [65]. The principal trade-off relative to FTRD is technical demand: classical EFTR requires advanced ESD competency, robust closure capability, and confident management of intra-procedural full-thickness defects. Where surgical alternatives such as laparoscopic wedge resection, LECS (laparoscopic–endoscopic cooperative surgery), or NEWS (non-exposed endoscopic wall-inversion surgery) are available, choice between approaches is increasingly individualised to lesion size, location, growth pattern, and the availability of multidisciplinary expertise.

### 7.4. Knife-Assisted Full-Thickness Resection (kFTR) of the Oesophagus

While FTRD and classical EFTR are predominantly applied to subepithelial lesions, knife-assisted full-thickness resection (kFTR) extends the EFTR principle to deeply invasive mucosal cancers in the oesophagus. The muscle-retracting sign, a dynamic endoscopic finding in which the submucosal dissection plane narrows due to tethering of the muscularis propria to the invasive tumour front, was first described in the colorectal setting as a predictor of deep invasion [58], and prospectively validated in rectal cancer [59]. Argenziano and Sorge et al. applied this sign to guide kFTR in the lower GI tract [66], establishing the pocket-guided technique before its translation to the oesophagus.

Oesophageal kFTR has been reported as a local control strategy in patients with locally advanced distal oesophageal adenocarcinoma (clinical stage T1b–T2) unfit for surgery [67]. A submucosal pocket is created cranial to the suspected invasive component; the muscle-retracting sign confirms the level of MP involvement; saline immersion with concurrent NGT drainage maintains optical clarity during peri-oesophageal dissection. In a case series of three patients, R0 for pT1b sm3 adenocarcinoma was achieved in 1/3 patients; oral intake resumed on day 5 without major adverse events [67]. Oesophageal kFTR provided tumour debulking and an intact histological specimen, offering potential advantages over palliative stenting alone. On current evidence, oesophageal kFTR should be regarded as an experimental technique, supported by a single three-patient case series with R0 achieved in 1/3. Its safety profile and clinical role beyond palliation need to be established through prospective evaluation in larger controlled cohorts.

## 8. Non-Curative ESD Resections

### 8.1. Definitions and Clinical Significance

Non-curative ESD is defined as an endoscopic resection that does not meet established histopathological criteria for curative resection, most commonly because of features associated with residual disease or lymph node metastasis, including deep submucosal invasion, lymphatic or vascular invasion, poorly differentiated or undifferentiated histology, or positive vertical margins. The depth threshold for “deep” submucosal invasion differs by histological context (Table 3): ≥200 µm below the muscularis mucosae for oesophageal SCC, and ≥500 µm for Barrett’s-associated adenocarcinoma and gastric cancer [2]. Traditionally, such findings mandated surgical oesophagectomy or gastrectomy. The risk of lymph node metastasis is estimated at 5–20% depending on risk factor combination [68,69], meaning that a substantial proportion of patients would have been cured by the endoscopic procedure alone.

### 8.2. Risk Stratification of Lymph Node Metastasis

**Oesophageal squamous cell carcinoma**. For oesophageal SCC, submucosal invasion depth is the dominant risk determinant. SM1 invasion (≤200 µm below the muscularis mucosae) carries substantially lower metastatic risk than SM2 or SM3 invasion, and selected SM1 cases with no additional risk factors may be candidates for surveillance rather than surgery [68]. A recent Japanese multicentre study with central pathological review of 422 endoscopically resected ESCCs (103 metastatic events) refined this framework by confirming depth of invasion (pT1b-SM2 vs. SM1: OR 2.72) and lymphovascular invasion as independent predictors of metastasis, while dimension, differentiation, and infiltrative growth pattern did not retain significance in multivariate analysis. Importantly, metastatic risk increased with both the number and submucosal depth of LVI foci, arguing against binary positive/negative reporting of this feature [70].

**Barrett’s-associated oesophageal adenocarcinoma**. Risk stratification for T1b Barrett adenocarcinoma has evolved substantially in recent years. Individual risk calculators incorporating submucosal invasion depth, lymphovascular invasion, differentiation grade, and resection margin status have been developed from multicentre cohort data, allowing probability-based estimation of lymph node metastasis risk at the individual patient level [69]. A retrospective comparative series found comparable cancer-specific outcomes between endoscopic surveillance and surgical resection in carefully selected patients with non-curative ESD for pT1b oesophageal adenocarcinoma [71]. An international multicentre retrospective study (N = 106, 11 centres) of radical endoscopic resection of high-risk T1 oesophageal adenocarcinoma found that among patients managed with surveillance (n = 80), lymph node metastasis developed in 6%, distant metastasis in 7%, and EAC-related mortality was 5%. Critically, among the 26 patients who underwent surgery, 19% had residual T1 EAC and 8% had lymph node metastasis in the surgical specimen confirming that a substantial proportion referred for surgery had been cured endoscopically and underwent oesophagectomy unnecessarily [72]. These data have provided the rationale for prospective evaluation of surveillance-based management.

**Early gastric cancer**. The distinction between absolute and relative non-curative criteria introduced in the Japanese Gastric Cancer Association guidelines and incorporated into the ESGE framework stratifies patients into those at sufficiently low metastatic risk to consider surveillance and those in whom additional treatment is warranted [2]. Risk quantification has been further refined by the eCURA scoring system. This validated multicentre tool assigns weighted scores to four independent predictors of lymph node metastasis after non-curative gastric ESD: lymphovascular invasion, tumour size > 30 mm, positive vertical margin, and deep submucosal invasion [2,73]. Patients are stratified into low (eCURA A), intermediate (eCURA B), and high (eCURA C) risk categories, with lymph node metastasis rates of approximately 2.5%, 6.7%, and 22.7% respectively. The eCURA system provides a practical framework for individualised multidisciplinary decision-making in cases where a single non-curative criterion would otherwise trigger automatic surgical referral.

### 8.3. Prospective Trial Evidence and Emerging Pathways

The PREFER trial (NCT03222635), a prospective international multicentre cohort registry across 19 hospitals in Europe and Australia, is evaluating surveillance as an alternative to oesophagectomy after radical resection of T1b and high-risk T1a oesophageal adenocarcinoma. Preliminary results presented at DDW 2025 demonstrated low recurrence rates after R0 endoscopic resection of high-risk T1 Barrett adenocarcinoma, supporting a strict endoscopic surveillance strategy [74]. These results require confirmation in the full prospective dataset but represent an important step toward evidence-based individualisation of post-ESD management.

For oesophageal SCC, the role of adjuvant treatment after non-curative ESD remains less defined outside Japan. The Ad-ESD trial (NCT04135664), a prospective RCT comparing adjuvant oesophagectomy versus definitive chemoradiotherapy for pT1b oesophageal SCC after ESD, is ongoing in Japan [75] and will provide the first randomised data comparing these strategies directly. Its results will be particularly relevant to Western practice, where definitive chemoradiotherapy is increasingly considered as an organ-preserving alternative to surgery in this setting.

A dedicated prospective registry or RCT for non-curative gastric ESD management, analogous to PREFER for oesophageal adenocarcinoma, is currently lacking, representing an important evidence gap given the frequency of gastric ESD, especially in Eastern practice.

**Table 3:** Comparison of curative criteria after ESD across the three early upper gastrointestinal cancer sites: ESGE versus Japanese guidelines, with site-specific future directions.

Site	ESGE Curative Criteria [2]	Japanese Guidelines (JES/JGCA) [76,77]	Future Directions
**Oesophageal squamous cell carcinoma**	- **Very-low-risk (curative) resection:** En bloc R0 resection. Histology no deeper than pT1a-m2. Well or moderately differentiated. No lymphovascular invasion (LVI) No further staging procedure or treatment is recommended. - **Low-risk (curative) resection:** En bloc R0 resection. pT1a-m3 or pT1b-SM1 (≤200 µm) Well to moderately differentiated No lymphovascular invasion LVI In these cases, particularly if the lesion is bigger than 20 mm, there is a real (albeit low) risk of LNM and complete staging is recommended, with the risk from further therapy being balanced against the risk of LNM, in a multidisciplinary discussion.	- **Curative resection:** En bloc R0 resection pT1a-EP or pT1a-LPM without LVI. For pT1a-MM or pT1b-SM, additional treatment should be considered. If pT1a-MM with vascular invasion or pT1b-SM, esophagectomy or chemoradiotherapy is recommended as additional treatment, without sufficient evidence to definitively prefer one over the other.	Ad-ESD RCT (NCT04135664) will compare adjuvant oesophagectomy with definitive chemoradiotherapy for pT1b SCC after non-curative ESD [75].
**Barrett-associated oesophageal adenocarcinoma**	- **Very-low-risk (curative) resection:** En bloc R0 resection. pT1a, well or moderately differentiated, no LVI. - **Low-risk (curative) resection:** En bloc R0 resection, pT1b (submucosal invasion ≤ 500 µm), well or moderately differentiated, no LVI, R0.	No separate validated curative category specific to Barrett-associated oesophageal adenocarcinoma. Evidence for Barrett adenocarcinoma in Japan is limited.	Individual risk calculators integrate depth, LVI, differentiation grade, and margin status, yielding patient-level LNM probability [69]. Comparative data show similar cancer-specific outcomes for surveillance versus surgery in selected non-curative pT1b cases without histological risk factors [71]. An 11-centre series of 106 patients found that 19% of those sent for surgery had no residual T1 EAC and 8% had nodal metastasis [72]. PREFER trial (NCT03222635) prospectively tests surveillance after radical R0 ER of low-risk and high-risk pT1b EAC. DDW 2025 interim data showed uneventful follow-up in most cases, particularly in low-risk pT1b EAC [74].
**Gastric adenocarcinoma**	- **Very-low-risk (curative) resection:** En bloc R0 resection. if intramucosal (pT1a), well or moderately differentiated, no LVI: any size when without ulceration, ≤30 mm when ulcerated. - **Low-risk (curative) resection:** well to moderately differentiated, pT1b SM1 (submucosal invasion ≤ 500 µm), ≤30 mm, no LVI, without ulceration. - En bloc R0 resection of a ≤20 mm gastric intramucosal poorly differentiated carcinoma, with no lymphovascular invasion or ulcer. - In these cases, there is a real (albeit low) risk of LNM, and complete staging is recommended, with the risk from further therapy (surgery) being balanced against the risk of LNM in a multidisciplinary discussion. Undifferentiated histology: ESGE remains more cautious than JGCA and handles such cases individually in expert centres.	**Japanese Gastric Cancer Association 7th edition curability classification:** **eCuraA—curative resection:** En bloc resection with HM0, VM0, Ly0, V0, fulfilling one of the following: (i) differentiated-type dominant, pT1a, UL0, any size; (ii) undifferentiated-type dominant, pT1a, UL0, ≤2 cm; (iii) differentiated-type dominant, pT1a, UL1, ≤3 cm; or (iv) differentiated-type dominant, pT1b-SM1, ≤3 cm. If the undifferentiated component exceeds 2 cm in category (i), or if an undifferentiated component is present in the submucosal invasive component in category (iv), the resection is classified as eCuraC-2. **eCuraB—curative resection after selected locally recurrent disease:** Applied to a locally recurrent lesion after initial endoscopic resection classified as eCuraC-1, when the lesion meets the relevant eCuraA criteria for differentiated-type pT1a UL1 ≤ 3 cm or pT1b-SM1 ≤ 3 cm, and the combined size of the recurrent lesion and initial ESD specimen is ≤30 mm. **eCuraC-1—non-curative resection with low lymph-node-metastasis risk:** Histologically differentiated-type lesion that otherwise fulfils criteria for eCuraA or eCuraB but was either not resected en bloc or has a positive horizontal margin. **eCuraC-2—non-curative resection:** Any eCuraC resection not meeting the definition of eCuraC-1. Gastrectomy with lymphadenectomy is considered the standard treatment after eCuraC-2.	The eCURA scoring system (distinct from the JGCA eCura classification) weights four predictors of LNM after non-curative ESD: LVI, tumour size > 30 mm, positive vertical margin, deep submucosal invasion. It stratifies patients into low (eCURA A, ~2.5% LNM), intermediate (eCURA B, ~6.7%), and high (eCURA C, ~22.7%) categories. The score supports individualised decisions in cases where a single non-curative feature would otherwise trigger automatic surgical referral [2,73]. A prospective registry or RCT analogous to PREFER for non-curative gastric ESD is missing. This is an important evidence gap given the volume of gastric ESD in Eastern practice.

Abbreviations: EAC, oesophageal adenocarcinoma; ER, endoscopic resection; ESD, endoscopic submucosal dissection; HM0, horizontal margin negative; JES, Japanese Esophageal Society; JGCA, Japanese Gastric Cancer Association; LNM, lymph node metastasis; LPM, lamina propria mucosae; LVI, lymphovascular invasion; MM, muscularis mucosae; SCC, squamous cell carcinoma; SM, submucosa; UL0/UL1, ulceration absent/present; VM0, vertical margin negative.

## 9. Training, Simulation, and Artificial Intelligence

The technical demands of ESD and related advanced resection techniques have historically limited widespread adoption outside dedicated referral centres, particularly in Western practice. Structured training pathways, validated simulation models, and artificial intelligence (AI) support are beginning to address this gap, each contributing to more reproducible skill acquisition and procedural quality.

### 9.1. Competency-Based Training Frameworks

ESD has a prolonged learning curve, with minimum volume thresholds of 30–80 procedures typically cited before expert-level outcomes are consistently achieved, though this varies substantially with case mix, supervision intensity, and prior ESD-adjacent experience [78]. Procedure volume alone, however, is an incomplete surrogate for competency: operators may reach the numerical threshold without achieving the technical precision that defines safe independent practice. A Delphi consensus established competency-based assessment milestones for gastric ESD, incorporating procedural metrics such as dissection speed, en bloc resection rate, and complication rate alongside process markers as objective endpoints [79]. These milestones have been incorporated into structured curricula including the ESGE training pathway, which defines proctored case requirements, formative assessment checkpoints, and criteria for supervised independence—a shift from volume-based to competency-based credentialing that supports more standardised and reproducible training outcomes [80]. Adoption in the West nevertheless faces specific structural barriers: lower per-centre case volumes, a limited pool of proctors, and fragmented trainee exposure mean that the supervised-case prerequisites and proctoring intensity defined in the ESGE pathway are harder to deliver than in high-volume Eastern centres, and credentialing frameworks remain heterogeneous between countries and institutions.

### 9.2. Simulation-Based Training

Simulation enables deliberate practice of specific procedural phases under supervised conditions without patient exposure, and several model types have been validated for ESD skills acquisition. Ex vivo porcine gastric models provide tissue fidelity closely approximating human gastric anatomy, with prospective studies demonstrating transfer of technical skills from simulated to clinical [81].

A sequential ex vivo-then-in vivo pathway has been formalised in structured hands-on courses and helps ESD trainees traverse the initial learning curve before supervised human practice, though animal-model training is constrained by cost, dedicated facilities, veterinary support, and ethical considerations for live procedures. In a research letter, Hadjinicolaou et al. evaluated untutored esophagogastric ESD training of an experienced therapeutic endoscopist [82]. The training consisted of an ex vivo porcine model and in vivo human upper GI cases with anatomic progression following intensive observation at a high-volume centre. By tracking the performance trajectory of a single endoscopist using key ESD performance indicators, they suggested that untutored oesophageal and gastric ESD training may be feasible for expert therapeutic endoscopists achieving minimum competency standards. However, this is not in line with the current ESGE curriculum for ESD training, recommending that at least the first 10 procedures in humans should be done under the supervision of an ESD-proficient endoscopist, as this minimises risks for patients [80].

Synthetic tissue phantoms based on polyvinyl alcohol hydrogel (EndoGel) or konjac-flour multilayer sheets (G-Master) provide reproducibility and standardisation that biological models cannot match, with the most advanced platforms enabling simulation of multiple anatomical gastric locations to familiarise trainees with difficult positions before clinical exposure [83]. There are still several key limitations. Firstly, the validation of transfer to clinical performance is only available for a minority of models. Secondly, standardised outcome metrics for simulation-based assessment are still lacking, representing an active area of research.

### 9.3. Artificial Intelligence in Endoscopic Resection

AI applications in ESD are advancing on several fronts. Automated surgical phase recognition systems achieve expert-level accuracy in identifying procedural phases in real time, with applications in quality metrics, trainee assessment, and adaptive intraoperative support [84]. AI-based systems for real-time vessel detection during submucosal dissection have demonstrated high sensitivity for submucosal vessel identification, with potential to reduce inadvertent vessel cutting and intraprocedural bleeding [85].

Robotic-assisted ESD platforms represent the most technologically advanced frontier. A human RCT comparing robot-assisted ESD to conventional ESD demonstrated equivalent oncological outcomes with potential ergonomic benefits and reduced operator tremor [86].

Translation of these tools into routine practice nevertheless faces regulatory and safety hurdles. Most resection-focused AI systems remain investigational, and the regulatory pathway for adaptive or continuously-learning algorithms—which may change behaviour after deployment—is still evolving under the CE-marking (EU Medical Device Regulation) and FDA frameworks, requiring prospective clinical validation and locked or version-controlled models before approval. Real-time intraprocedural decision support also raises the need for continuous safety monitoring, human oversight to mitigate automation bias, and clear accountability when AI guidance is integrated into the resection workflow.

Looking forward, integration of these tools into unified platforms could shift AI in ESD from isolated aids to coherent procedural augmentation.

## 10. Conclusions

Endoscopic resection of early upper gastrointestinal cancer is advancing across several domains. Enhanced endoscopic imaging now supports accurate lesion detection and characterisation. A new generation of therapeutic endoscopes bridges the longstanding gap between diagnostic-grade optics, the working channel, and manoeuvrability.

Resection itself has become more refined. Traction is supported by randomised and meta-analytic evidence, and adaptive multipoint systems extend the principle further. Saline immersion offers clear optical and haemostatic advantages when isotonic fluid is used.

Closure technologies now cover the full range of post-ESD defects. Deep resection strategies, including STER, FTRD, kFTR, and, more recently, EID, are redefining endoscopic resection and organ preservation, though the evidence remains early-stage.

The management of non-curative resection is shifting toward structured risk stratification and, where appropriate, surveillance rather than surgery. Training, simulation, and emerging AI tools will determine how widely these advances reach beyond referral centres.

Together, they establish endoscopic resection as a minimally invasive, organ-preserving alternative to surgery across a growing range of early upper gastrointestinal cancers.

## Figures and Tables

**Table 1:** Image-enhanced and magnification modalities relevant to upper gastrointestinal ESD: principal role and strength of supporting evidence.

Modality	Principal Role in the ESD Workflow	Highest Level of Evidence in UGI	Key References
White-light HD endoscopy	Baseline lesion detection and extent assessment	Reference standard	—
Narrow-band imaging (NBI), incl. magnifying NBI	Detection and characterisation of oesophageal SCC and gastric neoplasia; margin/depth	RCTs and meta-analyses	[7,8,9,10,13,16,17]
Linked colour imaging (LCI)	Detection of early gastric cancer and intestinal metaplasia	RCT and meta-analysis	[4,5]
Blue-light/laser imaging (BLI)	Real-time detection of early gastric cancer	RCT	[6]
i-Scan/i-Scan optical enhancement (OE)	Detection and characterisation of early gastric cancer	Meta-analysis of observational data	[11,12]
Magnifying endoscopy with validated classifications (JES, MESDA-G)	Characterisation and prediction of invasion depth	Meta-analyses and validated classifications	[13,14,15,16,17]
Optical enhancement + magnification	Detection of intestinal metaplasia	Observational	[20]
Dual-focus/extended depth-of-field optics	Close-range characterisation and intraoperative margin assessment	RCT (dual-focus)	[19]
Amber-red imaging	Recognition of anatomical structures in third-space endoscopy	Prospective video study (early)	[18]

**Table 2:** Summary of endoscopic resection innovations: indications, strengths, and limitations.

Technique	Principal Indications	Strengths	Limitations	Evidence Level
**TRACTION AND IMMERSION STRATEGIES**				
**SITE** *Saline immersion therapeutic endoscopy*	UGI and colorectal ESD; fibrotic lesions, Barrett’s oesophagus ESD, lesions with poor gravity-assisted exposure	Buoyancy reduces need for traction Improved optical clarity Haemostatic pressure effect Enhanced electrosurgical conduction vs. water Eliminates CO_2_ smoke	Fluid accumulation in oesophagus requires drainage strategy Requires isotonic saline (not water) Equipment setup more complex	*Cohort series* *No RCT yet*
**Clip-with-line traction** *Single-point traction (incl. S-O clip, SLC)*	Oesophageal and gastric ESD as adjunct to standard technique	Reduces procedure time Improves en bloc rate Reduces perforation rate Low cost; widely available components	Single fixed attachment point Traction force diminishes as dissection progresses Requires repositioning for changing directions	*RCTs (CONNECT-E/G)* *Meta-analyses*
**REACT/ATRACT** *Adaptive multipoint countertraction devices*	Colorectal ESD (primary evidence); early UGI applications reported	Adjustable, distributed countertraction ATRACT: increasing force as dissection advances REACT: low cost, widely available materials Compatible with SITE	UGI evidence limited to case reports/small series Additional setup step Learning curve for device placement	*Prospective cohort* *Meta-analysis (rubber band)*
**Water pressure method**	ESD with severe fibrosis from prior treatment; submucosal plane difficult to expose	Enables dissection in fibrotic planes where standard ESD fails Salvage option for previously untreatable recurrent lesions	Requires general anaesthesia Learning curve for application Technically demanding Applicable only to select fibrotic cases	*Case series*
**DEEP AND FULL-THICKNESS RESECTION STRATEGIES**				
**EID** *Endoscopic intermuscular dissection*	Gastric lesions with superficial muscularis propria involvement not amenable to standard ESD	Organ-preserving alternative to full-thickness resection Novel anatomical plane extending endoscopic reach Muscle-retracting sign as intraoperative landmark	Very early-stage; evidence from originator case reports only No established patient selection criteria Long-term oncological outcomes unknown Requires expert-level ESD competency	*Case reports (2024–2026)* *Originator groups only*
**STER** *Submucosal tunnelling endoscopic resection*	MP-layer subepithelial lesions of oesophagus and proximal stomach ≤ 35 mm; intraluminal/transmural growth; oesophageal leiomyomas	En bloc resection of MP tumours without full-thickness breach Intact mucosal entry site prevents contamination Superior to VATS for oesophageal leiomyomas Durable long-term recurrence-free survival	Limited to oesophagus, cardia, fundus Size ceiling ~35 mm Not suitable for predominantly extraluminal growth Tunnel construction impractical in distal stomach	*Systematic review > 2900 patients* *Comparative series*
**FTRD** *Full-thickness resection device*	Small (<20 mm) antral/distal gastric SELs; non-lifting adenomas; scarred/recurrent lesions; duodenal adenomas and NETs	Simultaneous resection and closure in single deployment Device-assisted; reproducible Avoids laparoscopy/GA for small lesions	Size ceiling ~20 mm Not usable in oesophagus (no serosa) Limited access to fundus/high body Risk of pyloric/duodenal stenosis if clip encroaches on lumen Lower R0 rate in duodenum	*International multicentre series*
**EFTR/kFTR** *Exposed endoscopic full-thickness resection/knife-assisted full-thickness resection*	Gastric SELs > 20 mm or outside FTRD anatomical reach	No size ceiling imposed by device Near 100% complete resection in expert gastric GIST series Entirely endoscopic management of intra-procedural perforation	Requires advanced ESD competency and closure expertise Deliberate full-thickness perforation; risk of contamination Not suitable for oesophagus Limited to high-volume expert centres	*Case series and retrospective studies*
**CLOSURE AND STRICTURE PREVENTION**				
**Mantis clip** *Through-the-scope closure*	Large post-ESD defects; perforations	Wide opening and tissue grasping for larger defects	Mantis: risk of superficial-only apposition if muscle not engaged bilaterally Costs	*Prospective series*
**X-tack™ (Endoscopic HeliX Tacking System)** *Through-the-scope suturing*	Large post-ESD defects; perforations	Scope withdrawal not needed Useful in difficult locations Good technical feasibility	May not achieve complete closure alone (additional clips or other tools are sometimes needed)	*Case reports*
**OTSC** *Over-the-scope clip*	Perforations; STER and POEM entry sites; fistulae; full-thickness defects	Full-thickness tissue compression Reliable transmural sealing Established safety profile	Bulky cap limits scope manoeuvrability during deployment Not repositionable after release Higher cost than standard clips	*Prospective studies;*
**OverStitch/SutuArt** *Endoscopic suturing systems*	Large/complex defects beyond clip capacity; POEM/STER entry sites; very large ESD defects	Full-thickness suturing of large defects SutuArt: through-the-scope, no cap required Reduced delayed bleeding vs. clips in large defects	OverStitch requires double-channel scope SutuArt: early-stage evidence Higher cost and complexity	*Meta-analyses (OverStitch)* *Pilot studies (SutuArt)*
**Stricture prevention** *Post-circumferential oesophageal ESD*	Oesophageal ESD > 75% luminal circumference	Oral prednisolone: strongest evidence, practical, low cost Biodegradable stents: mechanical dilation and dissolution Cell sheet transplantation: dramatic reduction in trials	Cell sheet transplantation: pioneer centres only; manufacturing not standardised Steroids carry systemic risk with prolonged courses No head-to-head RCT comparing all three strategies	*Network meta-analysis* *Cell sheets: single landmark study*

Abbreviations: EID, endoscopic intermuscular dissection; ESD, endoscopic submucosal dissection; EFTR, endoscopic full-thickness resection; FTRD, full-thickness resection device; GIST, gastrointestinal stromal tumour; kFTR, knife-assisted full-thickness resection; MP, muscularis propria; NET, neuroendocrine tumour; OTSC, over-the-scope clip; POEM, peroral endoscopic myotomy; REACT, Rubber band-assisted ESD with Adjustable Countertraction; ATRACT, Adaptive TRACtion; RCT, randomised controlled trial; SEL, subepithelial lesion; SITE, saline immersion therapeutic endoscopy; STER, submucosal tunnelling endoscopic resection; UGI, upper gastrointestinal; VATS, video-assisted thoracoscopic surgery.

## Data Availability

No new data were created or analysed in this study.
